# Single-Laboratory Validation for the Determination of Cocoa Flavanols and Procyanidins (by Degree of Polymerization DP1–7) in Cocoa-Based Products by Hydrophilic Interaction Chromatography Coupled with Fluorescence Detection: First Action 2020.05

**DOI:** 10.1093/jaoacint/qsaa132

**Published:** 2020-09-26

**Authors:** Ugo Bussy, Gregory Hewitt, Yusuf Olanrewaju, Brian R May, Nicholas Anderson, Javier I Ottaviani, Catherine Kwik-Uribe

**Affiliations:** 1 Mars, Incorporated, 6885 Elm St, McLean, VA 22101, USA; 2 University of California Davis, Department of Human Nutrition, CA 95616-5270, USA

## Abstract

**Background:**

Flavanols and procyanidins are complex bioactives found in many foods such as cocoa. As their consumption is associated with health benefits, cocoa flavanols and procyanidins are receiving increasing attention from consumers, industry, researchers, and regulators.

**Objective:**

The objective of this study is to validate a method using hydrophilic interaction chromatography (HILIC) with fluorescence detection (FLD) and a commercially available reference material for the determination of flavanols and procyanidins (CF) in cocoa-based products.

**Methods:**

Method performances were evaluated for cocoa matrices with CF content that ranged from 0.8 to 500 mg/g, which included low CF matrices (milk and dark chocolate, cocoa powder, and liquor) and high CF matrices (cocoa extract and dietary supplement products). The method was validated in a single-laboratory by determining sensitivity, selectivity, linearity, stability, robustness, accuracy, and precision for each of the matrices.

**Results:**

The method was validated for cocoa matrices with CF content that ranged from 0.8 to 500 mg/g. Accuracy ranged from 86 to 99% and repeatability (RSD_r_) from 1.5 to 8.6% for CF.

**Conclusions:**

Analytical performances acquired through this single-laboratory validation study for a wide range of cocoa-based matrices demonstrate that this method is fit-for-purpose for the determination of flavanols and procyanidins in cocoa-based products.

**Highlights:**

Hydrophilic interaction chromatography (HILIC) with fluorescence detection was successfully used to determine total CF content in multiple product types. Single-laboratory method validation results demonstrate that the method is fit for purpose for cocoa-based matrices containing <0.8 to 500 mg/g of CF.

Flavanols and their related oligomers, the procyanidins, are a specific class of flavonoids present in many foods such as cocoa (1, 2), green tea (3, 4), apples (5), and grapes (6, 7), and within these foods, flavanols and procyanidins exist as complex and diverse structures. In the context of cocoa, there is a growing body of evidence that associates the regular consumption of flavanols and procyanidins from cocoa, collectively referred to as cocoa flavanols (CF), with a range of health benefits, notably cardiovascular benefits (8–11). Consequently, there is rising interest from consumers, food industry, and regulators in knowing the CF content of cocoa-based materials and consumer products (12, 13). In addition, a critical component further defining CF health benefits resides in researchers’ ability to access reliable assay methods. Unfortunately, the diversity of flavanols and procyanidins and complexity of their structures make the conventional determination of CF using pure reference standards not technically achievable. In addition, the isolation and characterization of procyanidin fractions to be used as primary standards in routine laboratory analysis remains cost prohibitive. One approach to making CF testing accessible and transferable involved the resolution of procyanidins according to their degree of polymerization using hydrophilic interaction chromatography, and via the development of relative response factors (RRFs) for quantification (14). These RRFs allowed for the establishment of calibration curves for procyanidins based on the commercially available reference standard for the cocoa flavanol monomer, (−)-epicatechin. As such, this approach bypassed the need for a calibrant for procyanidins, making the method economically accessible and transferable. Unfortunately, the use of RRFs is extremely sensitive to changes in experimental conditions and limits further method development and adaptation to new cocoa-based matrices. Additionally, migration to HPLC or UPLC hardware with different dwell volumes impacts the accuracy of the pre-defined and method-specific RRFs, making transitioning between systems impossible. This is attributed to the disparity in equilibration time and solvent composition at the time of elution from the column/detection, which impacts fluorescence response. In recent years, the RRF approach has been further jeopardized when the column supporting this RRF-based methodology showed performance issues that put CF testing at risk; among 75 columns purchased in 2017, 94% were deemed unsuitable for use in CF analysis (data not shown). These factors highlighted the need for a new method independent from the use of RRF with a defined single column. Figure 1.

**Figure 1.**
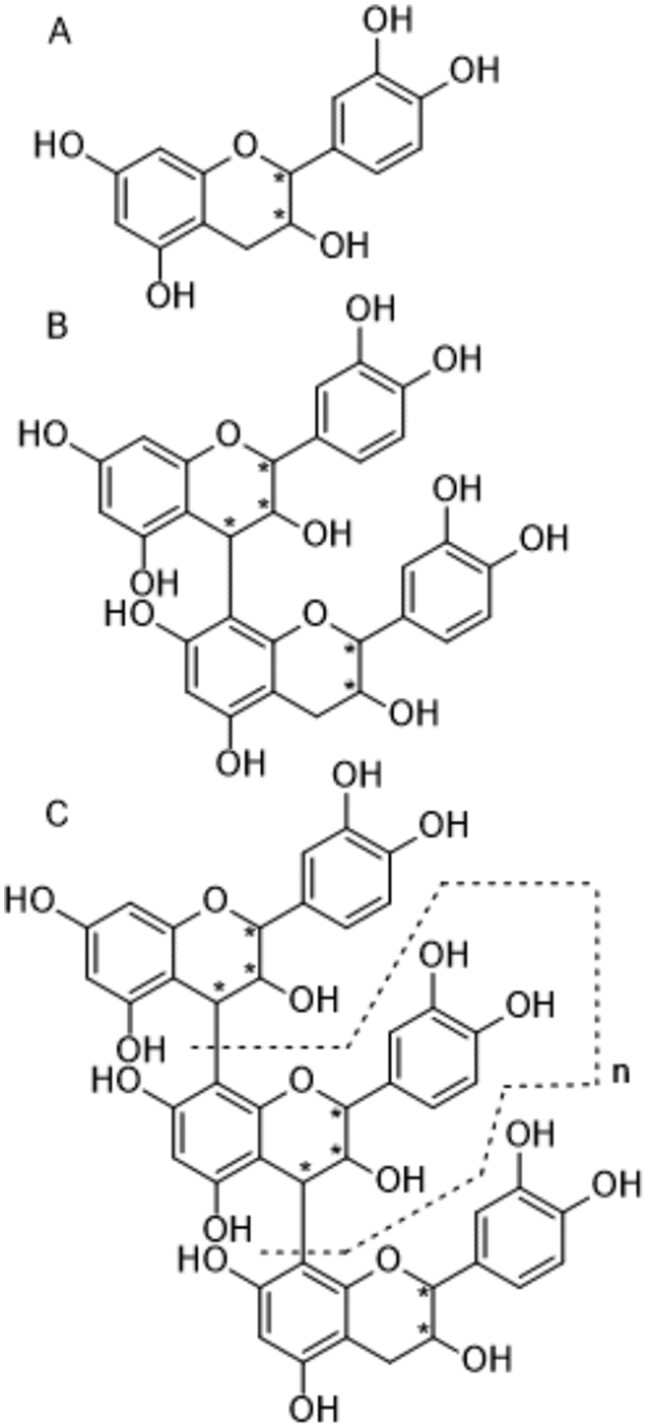
Chemical structure of cocoa flavanols and procyanidins. * highlight stereogenic atoms. A: cocoa flavanols monomer, B: b-type procyanidins, dimer and C: b-type procyanidin, trimer to heptamer (n=1-5).

The availability of a reference material for cocoa-specific flavanols and procyanidins (RM8403) by the National Institute of Standard and Technology (NIST, Gaithersburg, MD) has unlocked a new horizon in the development of analytical methodology for the reliable and accessible determination of CF. Specifically, this standard has allowed for the development and validation of a new, more robust method for a diverse set of cocoa-based products across a wide range of CF concentrations that could be readily and reliably transferred to other laboratories (15). End-user feedback was collected, leading to the development of thorough system suitability and improved sample preparation with the introduction of solid phase extraction clean-up and extension to matrixes with lower CF content. This updated method was implemented and validated for seven matrices as described in this single-laboratory validation (SLV) study against criteria defined by AOAC *Standard Method Performance Requirement* (SMPR^SM^) 2012.001.

## Validation Study Design

Method performances were assessed for seven matrices, namely milk chocolate, baking chocolate, cocoa liquor, cocoa powder, dietary supplement drink mix, dietary supplement capsules, and cocoa extract. Table 1 shows the CF compositions and concentration in each matrix (edible portion), as well as in the test sample (after preparation). A full method validation was carried out including the determination of specificity, accuracy, precision, linearity, sensitivity, robustness, and stability. These parameters were estimated for individual oligomeric fractions (Degree of Polymerization, DP, 1 through 7) and total CF (sum of DP1–7 contents). Accuracy was estimated by standard addition of reference material (NIST RM8403). Precision was estimated as Repeatability (RSD_r_) and assessed across triplicate preparation of a sample (after removal of the hexane soluble fraction) and was defined as the relative standard deviation of the concentration of each individual cocoa flavanol and procyanidin component and of total CF. Accuracy and precision experiments were implemented by two analysts using two HPLC systems and columns. Specificity was assessed through the comparison of retention time of each individual cocoa flavanol and procyanidin in the matrix of interest and the reference material. No significant retention time differences (<1%) were observed for the seven matrices studied. Linearity was assessed through the coefficient of determination for individual cocoa flavanol and procyanidin calibration curves. Coefficients of determination were systematically determined equal to or greater than 0.99. Limits of quantitation (LOQ) were determined as the ratio of 10 times the standard deviation of the signal area to the slope of the calibration curve for each of the 7 targets in each matrix. Sample stability was determined under analysis conditions (autosampler temperature 5°C) and under storage conditions (freezer temperature –18°C). Robustness was evaluated with deliberate changes made to mobile phase compositions (changing composition of acetic acid (2 ± 0.1%), water (3 ± 0.1%), column temperature (50 ± 1°C), and flow rate (1.00 ± 0.01 mL/min).

**Table 1.** Cocoa flavanol and procyanidin content (%) in cocoa-based matrices

	Cocoa flavanol and procyanidin content (%) in edible portion								Concentration	
	DP1	DP2	DP3	DP4	DP5	DP6	DP7	Total CF (DP1–7)	mg/g in edible portion	mg/mL as prepared
Extract	11.5	8.26	8.74	7.40	6.27	4.84	3.67	50.0	500	0.05
Capsules	10.3	7.43	7.87	6.66	5.64	4.36	3.30	45.0	450	0.05
Drink mix	2.41	1.73	1.84	1.55	1.32	1.02	0.77	10.5	105	0.07
Liquor	0.30	0.21	0.23	0.19	0.16	0.13	0.10	1.3	13	0.08
Cocoa powder	0.28	0.20	0.21	0.18	0.15	0.12	0.09	1.2	12	0.07
Baking chocolate	0.16	0.12	0.12	0.10	0.09	0.07	0.05	0.7	7	0.04
Milk chocolate	0.02	0.01	0.01	0.01	0.01	0.01	0.01	0.1	0.8	0.05

## AOAC *Official Method^SM^* 2020.05 Flavanol and Procyanidin (by Degree of Polymerization 1–7) of Cocoa Based Products  HILIC High-Performance Liquid Chromatography First Action 2020

[Applicable for the determination of flavanol and procyanidin content (DP 1–7) of cocoa-based matrices. The sum of monomeric (DP 1) and oligomeric fractions (DP 2–7) is reported as the total flavanol and procyanidin content.]

*Caution:* Solvents used are common-use solvents and reagents.

*Acetonitrile*.—Highly flammable, toxic, liquid irritant. Store in flammable liquid storage cabinet. Harmful if inhaled, swallowed, or absorbed through the skin. Use appropriate personal protective equipment and engineering controls, such as a laboratory coat, safety glasses, rubber gloves, and a fume hood. Dispose of acetonitrile and solutions according to federal, state, and local regulations.

*Glacial acetic acid*.—Corrosive, flammable liquid. Store in an acid storage cabinet. Causes severe burns. Use appropriate personal protective equipment and engineering controls, such as a laboratory coat, safety glasses, face shield, heavy rubber gloves, and a fume hood, when working with concentrated solutions. Dispose of acid and solutions according to federal, state, and local regulations.

*n-Hexane*.—Flammable, toxic, liquid irritant. Store in a flammable liquid storage cabinet. Harmful if inhaled, swallowed, or absorbed through the skin. Use appropriate personal protective equipment and engineering controls, such as a laboratory coat, safety glasses, rubber gloves, and a fume hood. Dispose of *n*-hexane and solutions according to federal, state, and local regulations.

*Methanol*.—Flammable, toxic, liquid irritant. Store in a flammable liquid storage cabinet. Harmful if inhaled, swallowed, or absorbed through the skin. Use appropriate personal protective equipment and engineering controls, such as a laboratory coat, safety glasses, rubber gloves, and a fume hood. Dispose of methanol according to federal, state, and local regulations.

*Acetone*.—Flammable, toxic, liquid irritant. Store in a flammable liquid storage cabinet. Harmful if inhaled, swallowed, or absorbed through the skin. Use appropriate personal protective equipment and engineering controls, such as a laboratory coat, safety glasses, rubber gloves, and a fume hood. Dispose of acetone and solutions according to federal, state, and local regulations.

## A. Principle

Chocolates, cocoa liquors, and cocoa powders are first extracted with hexane to remove their lipid components prior to extraction of flavanols and procyanidins. Flavanols and procyanidins (DP 1–7) are then extracted from these defatted materials and directly from cocoa extracts with an acidified aqueous acetone solvent system (acetone–water–acetic acid; AWAA). Finally, the extracts are cleaned up when necessary through solid phase extraction or filtered and transferred to chromatography vials for HILIC HPLC analysis. This extraction procedure is highly effective, reproducible, and does not result in loss or destruction of DP 1–7.

## B. Apparatus

- *HPLC system*.—Supporting back pressure of 400 bar, thermostated column compartment, solvent degasser, autosampler with temperature control, and fluorescence detector: Waters ACQUITY H-Class (Waters Corp., Milford, MA, USA), Agilent 1200/1260/1290, or similar.
- *Chromatography data acquisition software*.—Agilent ChemStation Plus Family, Revision C.01.09, Waters Empower 3 CDS, or equivalent.
- *HPLC column*.—Torus Diol 100 × 3.0 mm id, 130 Å, 1.7 µm particle size (Waters, Cat. No. 186007611), or equivalent.
- *Sonic bath*.—Capable of sonication and heating to at least 50°C (VWR, West Chester, PA; Model 150D), or equivalent.
- *Volumetric flasks*.—5, 10, 25, 50, or 100 mL.
- *Syringe filters*.—PTFE, 0.45 µm, 13 mm (Nalgene, Rochester, NY; Cat. No. 187-1345), or equivalent.
- *HPLC vials/caps*.—VWR (Cat. No. 608216-1232), or equivalent.
- *SPE cartridges.—*MCX PRiME, 30 µm, 150 mg/6cc (Waters; Cat. No. 186008919).
- *Vacuum manifold*.—24 position (Phenomenex; Cat. No. AH0-6024), or equivalent.
- *Syringes.—*3 mL (VWR; Cat. No. BD309586), or equivalent.
- *Disposable centrifuge tubes*.—15 and 50 mL (VWR; Cat. Nos 21008-210 and 240), or equivalent.
- *Centrifuge*.—Capable of 1700 rcf. (Sorval RC33 plus), or equivalent.
- *Vortex mixer*.—Fisher Scientific (Cat. No. 02-215-365), or equivalent.
- *Analytical balance*.—Readability to 0.1 mg.
- *Graduated cylinder*.—Fisher Scientific (Cat. No. 08552-4F), or equivalent.
- *Vacuum pump.—*Fisher Scientific (Cat. No. 16-108-554), or similar.

## C. Reagents

- *Water*.—Millipore quality (EMD Millipore, Billerica, MA), or equivalent.
- *Hexanes*.—HPLC grade (Fisher Scientific; Cat. No. H303-4), or equivalent.
- *Methanol*.—HPLC grade (Fisher A454-4), or equivalent.
- *Acetone*.—HPLC grade (Fisher A929-4), or equivalent.
- *Acetonitrile*.—HPLC grade (Fisher A998-4), or equivalent.
- *Acetic acid*.—Glacial (Mallinckrodt Baker, Inc., Phillipsburg, NJ; Cat. No. 9534-33), or equivalent.
- *Calibration standard*.—Cocoa extract reference material (NIST RM#no. 8403), or equivalent. Purity as indicated on the certificate of analysis.
- *Extraction solution*.—AWAA. Combine 700 mL acetone, 300 mL purified water, and 10 mL glacial acetic acid (70 + 30 + 1). Solution mixture referred to as AWAA is used for calibration standards, as well as for extraction of flavanols and procyanidins from test samples.

## D. System Conditioning and Suitability

FLD performance varies from manufacturer to manufacturer and even within instruments from same manufacturer. In order to quantitatively measure monomers (DP 1) through heptamers (DP 7) in a single measurement, the dynamic range must be optimized. Photomultiplier tube (PMT) gain setting for monomer (DP 1) needs to be established to ensure linearity of the signal, yet still retain maximum sensitivity for heptamer (DP 7), typically present in much lower quantity in cocoa-based materials. The instructions in this *System Suitability* section guide this dynamic range optimization process.

- *FLD sensitivity/dynamic range optimization and reproducibility*.—(1) Prepare a stock solution of cocoa extract reference material in AWAA at 0.2 mg/mL by weighing accurately 20 mg cocoa extract reference material into a 100 mL volumetric flask. Dilute to volume with AWAA solution. Prepare fresh. Do not store.
  (2) Select an appropriate starting sensitivity level (i.e., gain setting) on the FLD of the HPLC system; often, one can begin at instrument default. Injection volume is 2 µL.
  (3) Inject 0.2 mg/mL working solution of cocoa extract reference material three times using the HPLC conditions specified in **H**.
  (4) Observe whether the DP1 peak is of normal shape and the detector is not saturated.
  (5) If DP1 peak shape is normal and on-scale, repeat steps [D(a)(4) and (5)] at the next most sensitive detector gain setting. Consider that you might have to reduce the gain setting, e.g., PMT from 14 to 13, to ensure the proper dynamic range and to be able to measure all procyanidins.
  (6) Continue this process until the most sensitive detector gain setting for 0.2 mg/mL cocoa extract reference material working solution has been identified.
  (7) Once the optimum gain settings are identified, performed three subsequent injections of cocoa extract reference material at 0.2 mg/mL. %RSD on DP1 signal area must be ≤2%.
- *System suitability for analysis.—*(1) Each sequence must include ten subsequent injections of check working standard at 0.1 mg/mL (preparation described in section **E**(**c**). The five first injections are not evaluated and used only to insure complete the equilibration of the column. The injections 6–10 are evaluated for system suitability.
  - The relative standard deviation on signal area for each degree of polymerization must meet the following acceptance criteria: %RSD ≤2% for DP1, ≤2% for DP2, ≤5% for DP3, ≤5% for DP4, ≤10% for DP5, ≤15% for DP6, and ≤15% for DP7.
  - The average total CF determined on check working standard (injections 6-10) must be ≥90% and ≤ 110% of the expected value (referring to working standard certificate of analysis).
  (2) Each sequence must include calibration curve levels 1–5 (preparation described in section **E**). Coefficient of determination (r^2^) must be ≥ 0.99 for each DP1–7.
  (3) Each sequence must include bracketing standard (check working standard) injection every ten sample injections and must be followed by a blank injection (AWAA) prior to additional sample analysis.
  - System drift is verified against check working standard injections 6–10. Acceptable performances are recoveries in the check working standard of ±5, 10, and 20%, respectively, for DP1-4, DP5, and DP6-7.
  - Retention time for each DP in check working standard injection must be within 10% of the average retention time determined across check working standard injections 6–10.

## E. Preparation of Reference Material Solutions

- *Stock solution of Reference Material*.—Weigh 20 mg cocoa extract reference material into a 100 mL volumetric flask and dilute to volume with AWAA. This will be the 0.2 mg/mL standard stock solution and will be used as the level 5 working standard.
- *Dilutions*.—Prepare the dilutions using AWAA solvent, following dilution scheme in Table 2020.05A (e.g., dilute the stock solution of cocoa extract reference material 2.0 mL into a 10 mL volumetric flask giving level 1 working standard at 0.04 mg/mL). Prepare fresh. Do not store. The concentration for each DP can be calculated at each level of the calibration curve following Equation 1.
  (1) $$C_{\mathrm{DPn}}(\frac{mg}{mL})\;=\frac{{\mathrm{weight}}_{\mathrm{cocoa}\;\mathrm{extract}\;\mathrm{reference}\;\mathrm{material}}\left(g\right)\times {DP}_{n}c\mathrm{ontent}\;\mathrm{reference}\;\mathrm{material}(\frac{mg}{g})}{V_{\mathrm{stock}\;\mathrm{solution}\;of\;\mathrm{reference}\;\mathrm{material}}\;(mL)}\times DF$$
- *Check working standard.*—Weigh 20 mg cocoa extract reference material into a 100 mL volumetric flask and dilute to volume with AWAA. Pipette 5 mL into a 10 mL volumetric flask and dilute to volume with AWAA. This will be the 0.1 mg/mL check working standard stock solution. Correct mass using purity of each DP1–7 following Equation 1.
- *Sequence table: blanks, standards, and test samples*.—Cocoa extract calibration solutions are run routinely prior to sample analysis. *See*Table 2020.05B for a typical sequence that includes running system suitability samples (level 3), calibration solutions (levels 1–5), and a check sample (level 3).

**Table 2020.05A.** Preparation of cocoa extract calibration solutions

Level	Cocoa extract calibrant stock solution, mL	Total vol., mL	Concn, mg/mL	Dilution factor (DF)
1	2.0	10	0.04	5
2	4.0	10	0.08	2.5
3	6.0	10	0.12	1.67
4	8.0	10	0.16	1.25
5	N/A	100	0.20	1

**Table 2020.05B.** Sequence of samples

No.	Sequence of sample types	Notes/comments
1	Blank	—
2	System suitability solutions x10	Check working standard
3	Calibration solutions	Working standard levels 1–5
	Blank	—
4	Test samples	10-Sample run (or practice samples)
5	One check sample	Check working standard
6	Blank	—

## F. Removal of Lipid Fraction

For sample types expected to contain more than 10% fat, weigh approximately 5 g sample into a labeled 50 mL disposable centrifuge tube. Fill tube(s) to the 45 mL mark with hexane and cap tightly. Vortex at least 1 min to facilitate complete dispersion. Place tube(s) into a sonic bath at 50°C and sonicate for 5 min. Remove centrifuge tubes from the sonic bath and centrifuge all tubes for 5 min at 1700 rcf. Decant the hexane phase into a pre-tared beaker. Repeat this procedure twice more, combining the hexane layers, so that the extraction has been performed a total of three times. Allow the residual solids and hexane layer to dry in an appropriate fume hood until there is no evidence of remaining hexane. Weigh the residual solids from the hexane layers. Calculate the % fat as the amount of residual solids from the hexane layer divided by the initial sample weight times 100%.

## H. Extraction of Flavanols and Procyanidins

Accurately weigh the appropriate amount of sample (defatted if appropriate as defined in section (**F**) into a 50 mL disposable centrifuge tube according to Table 2020.05C. Accurately add the appropriate amount of AWAA. Hand-shake briefly, vortex as needed, until all solid is wetted to facilitate dispersion. Place sample tubes into a 50°C sonic bath for 5 min. Centrifuge all tubes for 5 min at 1700 rcf.

**Table 2020.05C.** Sample amount for extraction process

	Weight, mg	Vol., mL	Sonication	Centrifuge	SPE	Dilution	Filtering	Dilution factor (DF)
Milk chocolate	2000	5	5 min at 50°C	5 min at 1700 rcf	MCX PRiME	25 mL	None	50
Baking chocolate	300	10	5 min at 50°C	5 min at 1700 rcf	MCX PRiME	25 mL	None	100
Cocoa liquor	260	10	5 min at 50°C	5 min at 1700 rcf	MCX PRiME	25 mL	None	100
Cocoa powder	500	10	5 min at 50°C	5 min at 1700 rcf	MCX PRiME	25 mL	None	100
Drink mix	120	25^a^	5 min at 50°C	5 min at 1700 rcf	none	1 in 10 mL	PTFE 0.45 µm	250
Capsules	32	20	5 min at 50°C	5 min at 1700 rcf	MCX PRiME	25 mL	None	200
Cocoa extract	50	50 (flask)	None	None	None	1 in 10 mL	PTFE 0.45 µm	500

a Drink mixes are designed to be water soluble. To enhance extraction recovery, drink mixes were first dissolved in 7.5 mL of HPLC water, sonicated, and then mixed with 17.5 mL of acetone–acetic acid (100:1.5).

Certain matrices do not require SPE cleanup (e.g., cocoa extract) and can therefore be diluted to the desired concentration after dissolution according to Table 2020.05C. Dilution and sample preparation steps provided in Table 2020.05C are recommendation and might need to be adjusted or changed based on sample formulation. After dilution, filter using a 0.45 µm PTFE syringe filter and transfer to a HPLC autosampler vial.

Unless demonstrated to be unnecessary for the matrix, clean up extraction solution using a SPE MCX PRiME cartridge. Sample cleanup eliminates the accumulation of matrix components on column which leads to poor analytical performance. Perform conditioning of the SPE bed with 2 mL AWAA on a vacuum manifold. Do not allow the packing bed to dry at any time prior to loading the sample. After conditioning the column with AWAA, place a 15 mL centrifuge tube in vacuum manifold to collect and load 2.5 mL supernatant (extraction) solution. Move extraction solution through cartridge at low flow rate until 1–2 mm remain on top of the sorbent. Load 6 mL of AWAA and slowly move through cartridge using vacuum, repeat this step once (total of 12 mL of AWAA). Remove the tube from the vacuum manifold and transfer content to a 2 5 mL volumetric flask, dilute with AWAA. Homogenize flask and transfer approximately 1 mL to a HPLC autosampler vial (no filtering is required after SPE clean up).

*Note:* Necessity for SPE cleanup and/or scale can be reconsidered pending demonstration that the elimination of the cleanup doesn’t impact method performances and the ability to demonstrate system suitability.

## I. HPLC Parameters

- *Column and autosampler conditions*.—The column is a Torus diol (100 × 3.0mm id, 1.7 µm, 130Å particle size). Hold the column temperature at 50°C. The flow rate is 1 mL/min, and typical injection volume is 2 µL. Set the autosampler to, and hold at, 5°C. Equilibration of the column with 50/50 solvent A/solvent B for at least 10 min prior to analysis may be needed.
- *Solvents and gradient*.—The mobile phase is a binary gradient (solvents A and B) consisting of (A) acetonitrile–acetic acid (98 + 2, v/v) and (B) methanol–water–acetic acid (95 + 3 + 2, v/v/v). The starting mobile phase condition is 0% B; hold isocratic for 0.37 min. Subsequently, ramp solvent B to 45% over 10.03 min and to 95% B 0.25 min thereafter. Hold at 95% B for 2.35 min prior to returning to starting conditions (0% B) over 0.10 min. Total run time is 13.10 min. Postrun equilibration is 3 min.
- *Fluorescence detection.—*Conduct fluorescence detection with an excitation wavelength of 230nm and emission wavelength of 321 nm. Set the PMT to a level established in **D**(**a**) prior to conducting analyses.

## J. Integration

**Figure 2.**
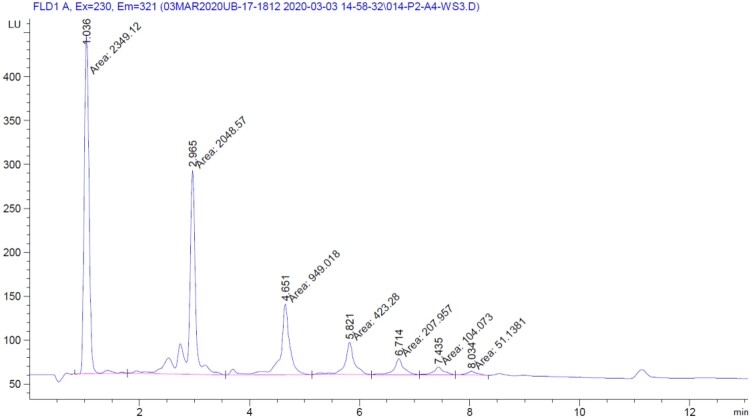
Sample HPLC trace showing DP 1–7. Sample was run with an Agilent 1290 system at a PMT gain setting of 13. Valley-to-valley integration is shown. The main point of this figure is to highlight integration format.

In the literature, two approaches for integration with HPLC methods have been reported, one integrating the complete baseline for the entire run, the other integrating the individual peaks valley-to-valley. R_s_ is clear for cocoa and chocolate samples, and a valley-to-valley integration approach was determined to be reproducible and robust in earlier method development steps (1). Additionally, since there is more than one species under each DP peak, and there can be moderate resolution of isomers of the procyanidins in one DP peak, providing visual guidance in the figure ensures reproducibility. *See*Figure 2 for an example.

## K. Quantification and Calculations

Cocoa extract reference material is used as the calibrant for all of the oligomeric fractions DP 1–7. Plot the concentration of each DP (mg/mL) from the standard solution concentrations on the *x*-axis and the FLD peak area on the *y*-axis. For each DP, calculate the resulting function from linear regression:

(2) $${\text{y}}_{\left(\text{DPn}\right)}={\text{ m}}_{\left(\text{DPn}\right)}x+\text{b}$$

where m is the slope and b is the *y*-value where the line intercepts the *y*-axis (*x *=* *0) obtained from running the Cocoa Extract Calibration solutions.

The sum of the quantities determined for each fraction (DP 1–7) is the total cocoa flavanol (CF; mg/g) content of the sample. Calculations are outlined in Equations 3 and 4 below.

Concentration of flavanols and procyanidins for individual fractions DP (*n*) is given in Equation 3:

(3) DPn CF mgg=areaDPn-bDPnmDPn×DF(sample weight (g)×(100-%fat100))

where CF_(DP__*n*__)_ = concentration, in mg/g, for one oligomeric fraction DP_*n*_ where *n* represents one of the oligomeric fractions (DP 1–7). The b_DPn_ and m_DPn_ values are determined from the calibration curve for that oligomer. Subsequent correction for the dilution factor (DF), sample weight (g), and fat content (%) are employed in the measurement. The calculation for each oligomeric fraction monomers through heptamers (DP 1–7) is required.

Concentration of total flavanols and procyanidin in defatted sample is given in Equation 4:

(4) $$\mathrm{Total}\;CF(mg/g)=\sum_{DP=1}^{7}CF\;(mg/g)$$

where Total CF is the concentration, in mg/g, of the sum of oligomeric fraction DP 1 (CF_(DP1)_) through DP 7 (CF_(DP7)_) in defatted sample.

## Results and Discussion

A SLV study was conducted with the objective to demonstrate method applicability to a wide range of CF concentrations and compositions of cocoa-based matrices. Previous studies focused on the determination of the sum of cocoa flavanols and procyanidins (16). The improvement in analytical instrumentation, the availability of new and higher performing chromatography columns, and the development of a reference material dedicated to cocoa flavanol and procyanidins now allows for the evaluation of method performances of monomeric cocoa flavanols and oligomers.

Sensitivities (LOQ) were determined for each oligomeric fraction in each matrix in sample and edible fraction (Table 2). The LOQs determined were systematically below the lowest point of calibration for cocoa extract. In addition, CF content ranging from 0.8 to 500 mg/g were determined successfully. SMPR 2012.001 defines the method operating range from 0.05 to 500 mg/g. The limit of quantification of 0.05 mg/g was not reached in this study because no cocoa-based matrices could be obtained at such low levels. To the best of our knowledge, milk chocolate products contain the lowest levels of CF and were successfully quantified using this method. The determination of CF in milk chocolate samples demonstrated the adequate performances of the method. Method specificity was confirmed by the relative difference of retention times of analytes in sample matrix to retention time of analyte in reference material solution. These differences were systematically below 1%.

**Table 2.** LOQ determined for each oligomer in each matrix expressed in µg/mL for samples (in vial) and in mg/g of edible portion

	LOQ, µg/mL in sample							LOQ, mg/g in edible portion						
	DP1	DP2	DP3	DP4	DP5	DP6	DP7	DP1	DP2	DP3	DP4	DP5	DP6	DP7
Extract	0.51	0.24	0.36	0.41	0.71	0.70	0.63	5.07	2.39	3.65	4.06	7.13	7.00	6.28
Capsules	0.32	0.21	0.89	0.72	0.71	0.80	0.46	1.99	1.33	5.59	4.51	4.46	5.01	2.90
Drink mix	0.26	0.72	0.67	0.45	0.70	0.21	0.32	0.54	1.50	1.39	0.93	1.46	0.44	0.66
Liquor	2.98	2.32	2.18	1.87	1.74	0.79	0.47	0.44	0.34	0.32	0.28	0.26	0.12	0.07
Cocoa powder	1.05	0.74	1.16	0.93	1.03	0.89	1.13	0.18	0.13	0.20	0.16	0.18	0.16	0.20
Baking chocolate	0.64	1.14	0.98	1.20	1.05	1.36	0.91	0.10	0.18	0.15	0.19	0.17	0.21	0.14
Milk chocolate	1.63	2.21	2.26	1.56	1.00	0.60	0.36	0.03	0.04	0.04	0.03	0.02	0.01	0.01

Linearity was estimated through the determination of the coefficient of determination for cocoa flavanol and each procyanidin calibration curve. Coefficient of determination were systematically determined equal to or greater than 0.99.

Method robustness was demonstrated through minor, but deliberate changes made to column oven temperature and compositions of the mobile phases. The alteration of mobile phase composition led to minor, but consistent retention time shifts (ranging from 0.04 to 1.44%), highlighting the method robustness to fluctuations in mobile phase composition. The fluctuation of column temperature (50 ± 1°C) and flow rate (1.00 ± 0.01 mL/min) did not lead to significant retention time shifts (<1%). Sample stability was also evaluated for key matrices and is summarized by Table 3. These results demonstrate sample stability in the autosampler (4 days) and prolonged stability in the freezer (9 days).

**Table 3.** Total cocoa flavanol and procyanidin stability in analysis and storage conditions as published (15)

	Sample recoveries, % compared to day 0			
	Autosampler		Freezer	
	4 days	9 days	4 days	9 days
Extract	96	89	97	94
Capsules	103	106	101	106
Drink mix	97	94	99	98
Cocoa powder	96	91	97	96
Dark chocolate	93	89	99	92

Accuracy and precision were thoroughly assessed for each oligomeric fraction in each of the seven matrices studied. Accuracy was assessed through a standard addition experiment using cocoa extract RM8403, while precision was assessed through the %RSD of levels determined in triplicate preparation of samples. Experiments were duplicated by a different analyst, instrument, column, date and sample preparation. Table 4 shows the % recoveries determined, which were all meeting or within 2% of SMPR 2012.001 performance requirements, except for the lowest concentration matrix, milk chocolate. Performance requirements are defined using content in edible fraction and can thus change between oligomer and matrices.

**Table 7.** Recovery (%) determined for individual for individual cocoa flavanol and procyanidin and total cocoa flavanol (DP1–7). Respective performance requirements documented in SMPR 2012.001 are listed for relevant concentration ranges

Recovery, %	DP1	DP2	DP3	DP4	DP5	DP6	DP7	Total CF (DP1–7)
Extract	98^b^	103^c^	102^c^	102^c^	102^c^	103^c^	97^c^	98^a^
Capsules	103^b^	102^c^	100^c^	100^c^	96^c^	91^c^	103^c^	95^a^
Drink mix	95^c^	101^c^	99^c^	99^c^	100^c^	98^c^	94^d^	96^b^
Liquor	100^d^	98^d^	97^d^	98^d^	99^d^	97^d^	96^d^	95^c^
Cocoa powder	101^d^	107^d^	101^d^	102^d^	103^d^	104^d^	101^e^	99^c^
Baking chocolate	93^d^	97^d^	95^d^	96^d^	94^e^	93^e^	92^e^	91^c^
Milk chocolate	95^e^	94^e^	92^e^	88^e^	81^e^	80^e^	88^e^	86^c^

a 98–101%.

b 95–102%.

c 92–105%.

d 90–108%.

e 85–110% based on content in edible fraction.

Precision was assessed through repeatability experiments and the determination %RSD across triplicate preparations (RSD_r_). Repeatability results are summarized in Table 5 and systematically meet performance requirements from AOAC SMPR 2012.001 (RSD_r_≤5%) for all but milk chocolate. This finding is consistent with the %recovery observed for milk chocolate. The relative lack of performance of the method for the milk chocolate matrix is associated with the very low CF content (<1 mg/g) rather than with method performances. Precision on individual oligomeric concentration was also assessed. SMPR 2012.001 performance requirements were met except for DP6-7 in cocoa liquor and DP7 in drink mix and baking chocolate, as well as DP5-7 in milk chocolate. However, the method precision on oligomeric content did not exceed 10% for cocoa liquor, 8% for baking chocolate, 7% for drink mix, and 13% for milk chocolate. Precision of the method was expected to decrease with increasing degree of polymerization because of lower concentration and broadened peak shape leading to measurement challenges.

**Table 5.** Repeatability (RSD_r_) determined for individual cocoa flavanol and procyanidin and total cocoa flavanol (DP1–7) for triplicate preparations in cocoa-based dietary supplements and foods. Respective performance requirements documented in SMPR 2012.001 are listed for relevant concentration ranges

Repeatability, RSD_r_	DP1	DP2	DP3	DP4	DP5	DP6	DP7	Total CF (DP1–7)
Extract	1.9 ^a^	2.1^a^	2.5^a^	2.9^a^	2.2^a^	3.3^a^	2.8^a^	2.3^a^
Capsules	2.5^a^	2.2^a^	2.1^a^	2.5^a^	2.8^a^	3.4	1.6^a^	2.3^a^
Drink mix	2.4^a^	2.5^a^	2.5^a^	3.6^a^	2.9^a^	2.2	6.7	1.9^a^
Liquor	3.7^a^	3.6^a^	3.2^a^	4.4^a^	5.0^a^	7.8	10.0	4.0^a^
Cocoa powder	1.5^a^	2.3^a^	2.6 ^a^	1.1^a^	1.6	0.4	4.4^b^	1.5^a^
Baking chocolate	1.9^a^	3.2^a^	2.6^a^	2.4^a^	3.6^a^	1.4^a^	7.4^b^	1.9^a^
Milk chocolate	5^b^	7^b^	9^b^	10^b^	11^b^	13^b^	13^b^	8.6 ^a^

a <6%.

b <10%, based on content in edible fraction.

In addition to this SLV, reproducibility data were acquired across multiple laboratories for most matrixes and have been reported in a previous study (12). Further cross-lab transferability will be verified in a future multi-lab validation. Reproducibility values are shown in Table 6 and are systematically within 2% of the performance requirements described by AOAC SMPR 2012.001. RSD_R_ were expected and measured higher than RSD_r_ but demonstrated method transferability with reproducibility on total cocoa flavanol not exceeding 10%.

**Table 6.** Reproducibility (RSD_R_) determined for individual cocoa flavanol and procyanidin and total cocoa flavanols in cocoa-based dietary supplements and foods and reported in (12).Reproducibility were assessed across four laboratories except for baking chocolate which was assessed across eight laboratories. Respective performance requirements documented in SMPR 2012.001 are listed for relevant concentration ranges

Reproducibility, RSD_R_	DP1	DP2	DP3	DP4	DP5	DP6	DP7	Total CF (DP1–7)
Extract	2.1^a^	4.4^a^	3.7^a^	2.4^a^	1.0^a^	4.3^a^	5.2^a^	2.6^a^
Capsules	9.2^a^	10.5^a^	9.0^a^	8.8^a^	8.7^a^	7.4^a^	5.1^a^	9.7^a^
Drink mix	9.2^a^	7.9^a^	8.0^a^	8.3^a^	7.6^a^	8.0^a^	8.3^a^	7.9^a^
Liquor	ND^a,b^	ND^a,b^	ND^a,b^	ND^a,b^	ND^a,b^	ND^a,b^	ND^a,b^	ND^a,b^
Cocoa powder	5.4^a^	10.5^a^	9.1^a^	7.2^a^	5.5^a^	4.0^a^	6.3^a^	6.2^a^
Baking chocolate	5.4^a^	7.7^a^	9.0^a^	6.0^a^	7.9^a^	11.8^a^	10.6^b,c^	6.9^a^
Milk chocolate	ND^b,c^	ND^b,c^	ND^b^	ND^b,c^	ND^b,c^	ND^b^	ND^b,c^	ND^b,c^

a <8%. ^b^ND = not determined.

b <18%, based on content in edible fraction.

## Conclusions

The range of cocoa-based matrices studied, and the evidence of analytical performances acquired through single-laboratory validation study reported in this article, in combination with previous evidence of reproducibility performance, demonstrate this method is fit-for-purpose for the determination of flavanols and procyanidins in cocoa-based products. Accuracy, precision, linearity, sensitivity, selectivity, stability, and robustness were assessed. Method accuracy was demonstrated adequate, with recoveries determined within 2% of the ideal performances summarized by AOAC SMPR 2012.001. Similarly, method precision was demonstrated appropriate, with repeatability determined within 5% of the ideal performances listed in AOAC SMPR 2012.001. As expected, it should be noted that the analysis of low CF contents (<10 mg/g) in matrices like chocolate led to lower performances.

The evaluation of reproducibility through the multi-laboratory validation and collection of end-user feedback during the 2-year first action period will further enhance method reliability and applicability to routine analysis.
